# Updated meta-analysis on intraoperative inspired fraction of oxygen and the risk of surgical site infection in adults undergoing general and regional anesthesia

**DOI:** 10.1038/s41598-023-27588-2

**Published:** 2023-02-11

**Authors:** Yoann El Maleh, Charlotte Fasquel, Christophe Quesnel, Marc Garnier

**Affiliations:** 1 Sorbonne University, GRC29, Assistance Publique-Hôpitaux de Paris (APHP), DMU DREAM, Anesthesiology and Critical Care Medicine Department, Tenon University Hospital, 4 rue de la Chine, 75020 Paris, France; 2 Centre Hospitalier Régional Universitaire de Brest, Service d’Anesthésie-Réanimation et Médecine Périopératoire, 29200 Brest, France

**Keywords:** Bacterial infection, Risk factors

## Abstract

This updated meta-analysis aims at exploring whether the use of systematic high vs low intraoperative oxygen fraction (FiO_2_) may decrease the incidence of postoperative surgical site infection during general (GA) or regional anesthesia (RA). PubMed, Cochrane CENTRAL, ClinicalTrials.gov databases were searched from January 1st, 1999 and July, 1st 2022, for randomized and quasi-randomized controlled trials that included patients in a high and low FiO_2_ groups and reported the incidence of SSI. The meta-analysis was conducted with a DerSimonian and Laird random-effects model. Thirty studies (24 for GA and 6 for RA) totaling 18,055 patients (15,871 for GA and 2184 for RA) were included. We have low-to-moderate-quality evidence that high FiO_2_ (mainly 80%) was not associated with a reduction of SSI incidence compared to low FiO_2_ (mainly 30%) in all patients (RR 0.90, 95%CI 0.79–1.03). Moderate inconsistency existed between studies (I^2^ = 38%). Subgroup analyses showed a moderate protective effect in patients undergoing GA (RR 0.86, 95%CI 0.75–0.99) (low level of evidence), while high FiO_2_ was not associated with a reduction of SSI in patients undergoing RA (RR 1.17, 95%CI 0.90–1.52) (moderate level of evidence). Sensitivity analyses restricted to patients ventilated without nitrous oxide (n = 20 studies), to patients operated from abdominal surgeries (n = 21 studies), and to patients suffering from deep SSI (n = 13 studies), all showed the absence of any significant effect of high FiO_2_. As a conclusion there is no compelling evidence that high FiO_2_ can improve postoperative patient’s outcome on its own when good SSI prevention practices are properly applied. Recent well-designed and adequately powered randomized controlled trials add further weight to these results.

## Introduction

Surgical site infections (SSI) are the most common healthcare-associated infections and a source of morbidity and over-mortality. In 2016, a systematic review and meta-analysis assessing the effects of systematic high FiO_2_ (80%) compared with standard FiO_2_ (30%) concluded that high FiO_2_ were associated with a reduction of SSI in patients undergoing surgery under general anesthesia^1^. Consequently, the WHO recommended that “adult patients undergoing general anesthesia should receive an 80% FiO_2_ intra-operatively to reduce the risk of SSI”^2^. These recommendations have sparked large debate on the benefits and harms of hyperoxemia. On the theoretical point-of-view, several pro (prevention of hypoxemia, SSI and postoperative nausea and vomiting) and con (respiratory adverse events, increased production of harmful “reactive oxygen species”) arguments have been raised by believers and detractors of high FiO_2_. Accordingly, and despite these recommendations, anesthetists still used a wide range of intraoperative FiO_2_ in daily practice^3,4^ and frequently changed FiO_2_ settings during surgery unrelated to patients’ PaO_2_ or SpO_2_^5^.

Then, an updated meta-analysis still reported in 2018 a beneficial effect of high FiO_2_, however with an increasingly low level of evidence^6^. Consequently, the WHO downgraded the strength of its recommendations but still recommended an 80% intraoperative FiO_2_ during general anesthesia^7^. In 2019, de Jonge et al*.* updated the meta-analysis in turn and reported a significantly lower incidence of SSI in intubated patients ventilated intra-operatively with high compared to low FiO_2_ (RR 0.80 [0.64–0.99])^8^. In the same issue of the *British Journal of Anesthesia*, Mattishent et al*.* reported the results of a meta-analysis focused on the safety of high FiO_2_, demonstrating the absence of significant side-effects, in particular regarding respiratory and cardiovascular events^9^. Several reasons can be suggested to explain persistent mistrust. First, de Jonge’s meta-analysis still included Myles’ ENIGMA study whereas it compared 80%O_2_/20%N_2_ to 30%O_2_/70%N_2_O and was originally designed to assess benefits and harms of nitrous oxide. Second, substantial heterogeneity remained, leading de Jonge and co-authors to conclude that “the evidence from the updated analysis has become weaker”. Third, this meta-analysis may have become obsolete very quickly, as new randomized studies were published in the following months. One year later, Hovaguimian et al. concluded in an updated meta-analysis of their former work of 2013, based on 8 studies, that the exclusion of the retracted studies by the group of Schietroma led to a “confidence interval [around the relative risk] wider, that now crossed the line of equality”^10^. However, despite this non-significant updated result, the authors concluded that “consistently with the WHO meta-analysis, high inspired oxygen could have a protective effect against surgical site infection”. It can be said that all this did not bring the debate to be closed.

The aim of this study was to update the meta-analysis on the potential beneficial effects of the administration of high intraoperative FiO_2_ on the occurrence of SSI.

## Results

### Description of the included studies

**Figure 1:**
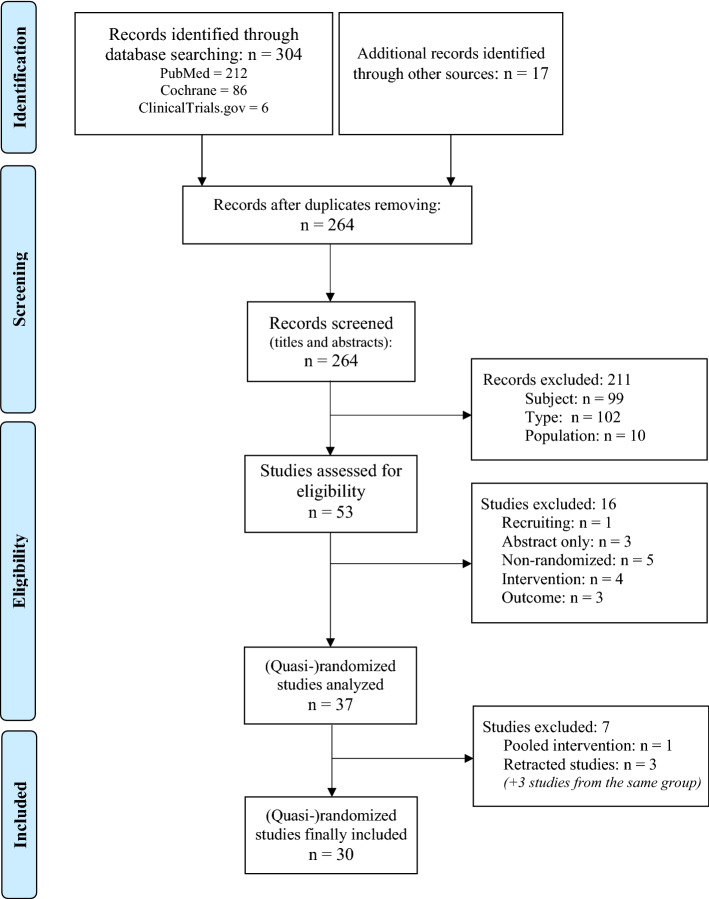
Flow diagram of study selection.

The main characteristics of the studies included in the meta-analysis are summarized in Table 1 (general anesthesia) and Table 2 (regional anesthesia). Thirty-seven studies were first included (Fig. 1). Then, 3 RCT by Schietroma et al*.* were excluded from analysis taking into account the retraction of 2 of them due to the falsification of the statistics^11,12^ and one of them for plagiarism and similarities of data with those previously published by another group^13^. The validity of the 3 non-retracted studies from this group^14–16^ has also been questioned because all 6 RCT of this group reported results markedly different from the pooled results of all other published trials systematically in favor of the high FiO_2_ group. Consequently, as previous authors^8–10^, we followed the conclusions of the extensive re-analysis of the whole work from Schietroma’s group^17^ and did not include data from any study of this group in our meta-analysis.

**Table 1:** Summary of the main characteristics and results of the studies including patients under general anesthesia.

Study	Country	Design, n	Type of surgery	O_2_ duration in PACU	SSI definition, follow up	SSI, n (%)	Second gas	Antibiotic prophylaxis	Temp	Fluids
Greif et al.^19^	Austria, Germany, USA	RCT Multicenter n = 500	Colorectal	2 h	Wound infection (pus), Day 15	FiO_2_ 80%: 13/250 (5.2%) FiO_2_ 30%: 28/250 (11.2%) *p* = 0.01	N_2_	*NP*	Yes	15 ml/kg/h
Pryor et al.^20^	USA	RCT Monocenter n = 160	Major abdominal laparotomy or laparoscopy	2 h	Clinical and paraclinical requiring medical support, Day 14	FiO_2_ 80%: 20/80 (25%) FiO_2_ 35%: 9/80 (11.3%) *p* = 0.02	N_2_O	Yes	*NP*	*NP*
Belda et al.^21^	Spain	RCT Multicenter n = 291	Colorectal laparotomy	6 h	CDC, Day 14	FiO_2_ 80%: 22/148 (14.9%) FiO_2_ 30%: 35/143 (24.4%) *p* = 0.04	Air	Yes	Yes	15 ml/kg/h
Mayzler et al.^22^	Israel	RCT Monocenter n = 38	Colorectal Carcinologic	2 h	Wound infection, Day 30	FiO_2_ 80%: 2/19 (12.5%) FiO_2_ 30%: 3/19 (17.6%) *p* = 0.53	N_2,_ N_2_O	Yes	Yes	15 ml/kg/h
Myles et al.^23^	Australia	RCT Multicenter n = 2012	Major surgery > 2 h	–	Wound infection (pus or positive culture), Day 30	FiO_2_ 80%: 77/997 (7.7%) FiO_2_ 30%: 106/1015 (10.4%) *p* = 0.034	N_2,_ N_2_O	Yes	*NP*	*NP*
Meyhoff et al.^24^	Denmark	RCT Multicenter n = 1386	Abdominal laparotomy	2 h	CDC, Day 14	FiO_2_ 80%: 131/685 (19.1%) FiO_2_ 30%: 141/701 (20.1%) *p* = 0.64	Air	Yes (70% of cases)	Yes	Restrictive
Bickel et al.^25^	Israel	RCT Monocenter n = 210	Appendectomy Mac Burney	2 h	ASEPSIS, Day 14	FiO_2_ 80%: 6/107 (5.6%) FiO_2_ 30%: 14/103 (13.6%) *p* = 0.04	N_2_ Air	Yes	Yes	*NP*
Thibon et al.^26^	France	RCT Multicenter n = 434	Abdominal laparoscopy/tomy + breast cancer surgery	–	CDC, Day 30	FiO_2_ 80%: 15/226 (6.6%) FiO_2_ 30%: 15/208 (7.2%) *p* = 0.81	Air	Yes (51.5% of cases)	*NP*	*NP*
Chen et al.^27^	Hong Kong	RCT Monocenter n = 91	Colorectal	24 h	CDC, Day 30	FiO_2_ 80% + N_2_: 5/30 (16.7%) FiO_2_ 30% + N_2_: 4/30 (13.3%) FiO_2_ 30% + N_2_O: 15/31 (48.4%) *p* = 0.21	N_2_ N_2_O	Yes	Yes	*NP*
Stall et al.^28^	USA	RCT Monocenter n = 235	Orthopedic trauma surgery	2 h	CDC, Day 84	FiO_2_ 80%: 14/119 (12%) FiO_2_ 30%: 19/116 (16%) *p* = 0.31	*NA*	Yes	*NP*	*NP*
Kurz et al.^29^	USA, Ireland, Austria	RCT Multicenter n = 555	Colectomy laparotomy > 2 h	1 h	CDC, Day 30	FiO_2_ 80%: 45/285 (15.8%) FiO_2_ 30%: 42/270 (15.6%) *p* = 1.00	N_2_	Yes	*NP*	6–10 ml/kg/h
Wasnik et al.^30^	India	RCT Monocenter n = 64	Appendectomy Mac Burney	2 h	ASEPSIS, Day 14	FiO_2_ 80%: 0/32 FiO_2_ 30%: 0/32	*NA*	Yes	*NP*	*NP*
Chiang et al.^31^	New Zealand	RCT Monocenter n = 37	Vascular surgery (infra-inguinal bypass)	2 h	CDC, Day 30	FiO_2_ 80%: 6/19 (incl. 0 major SSI) FiO_2_ 30%: 7/18 (incl. 3 major SSI) *p*> 0.05	*NA*	Yes	Yes	*NP*
Kurz et al.^32^	USA	Quasi-randomized Monocenter n = 5749	Major abdominal > 2 h laparotomy or laparoscopy	–	CDC, Day 30	FiO_2_ 80%: 118/2896 (4.1%) FiO_2_ 30%: 112/2853 (3.9%) *p* = 0.77	*NA*	Yes	Yes	*NP*
Mayank et al.^33^	India	RCT Monocenter n = 94	Colorectal	6 h	CDC, Day 30	FiO_2_ 80%: 26/47 (55.3%) FiO_2_ 30%: 19/47 (40.4%) *p* = 0.21	N_2_O	Yes	Yes	15 ml/kg/h
Alvandipour et al.^34^	Iran	RCT Monocenter n = 80	Colorectal	1 h	ASEPSIS, 1 month following discharge	FiO_2_ 80%: 2/40 (5%) FiO_2_ 30%: 6/40 (15%) *p*< 0.05	N_2_O	Yes	Yes	6–10 ml/kg/h
Ferrando et al.^35^	Spain	RCT Multicenter n = 717	Abdominal > 2 h	3 h	CDC, Day 7 (main outcome) and day 30	FiO_2_ 80%: 31/362 (8.9%) FiO_2_ 30%: 34/355 (9.4%) *p* = 0.90 FiO_2_ 80%: 52/362 (16.5%) FiO_2_ 30%: 62/355 (19.9%) *p* = 0.89	Air	Yes (85% of cases)	*NP*	*NP*
Li et al.^36^	China	RCT Monocenter N = 251	Abdominal > 2 h	2 h	CDC, Day 7	FiO_2_ 80% 12/126 (9.5%) FiO_2_ 30%: 18/125 (14.4%) RR: 1.51, *p* = 0.23	Air	Yes	Yes	8 ml/kg/h
Lin et al.^38^	China	RCT Monocenter N = 630	Abdominal carcinologic 2–5 h	–	Wound infection Delay ?	FiO_2_ 80% 40/316 (12.7%) FiO_2_ 40%: 30/314 (9.6%) *p* = 0.74	Air	Yes	Yes	*NP*
Park et al.^37^	Korea	RCT Monocenter N = 172	Abdominal surgery	15 min	Wound infection requiring re-intervention, During hospitalization	FiO_2_ 60%: 8/87 (9%) FiO_2_ 35%: 4/85 (5%) *p* = 0.25	Air	Yes	*NP*	*NP*
Yerra et al.^39^	India	RCT Monocenter N = 178	Emergency abdominal surgery	2 h	CDC + microbiological culture	FiO_2_ 80%: 29/85 (34.1%) FiO_2_ 30%: 19/93 (20.4%)	Air	Yes	*NP*	*NP*
Reiterer et al. ^40^	Austria	RCT Monocenter N = 258	Major abdominal surgery > 2 h	2 h	CDC	FiO_2_ 80%: 20/128 (15.6%) FiO_2_ 30%: 23/130 (17.7%) *p* > 0.05	*NA*	*NA*	*Yes*	2–3 ml/kg baseline + bolus guided by oeso. doppler
Major Extremity Trauma Research Consortium^41^	USA	RCT Multicenter N = 1136	Orthopedic trauma surgery	2 h	CDC, Day 182	FiO_2_ 80%: 40/575 (7.0%) FiO_2_ 30%: 60/561 (10.7%) RR : 0.65 [0.45–0.96]—*p* = 0.03	*NA*	Yes	*NP*	*NP*
Holse et al.^42^	Denmark	RCT Multicenter N = 576	General surgery > 1 h	2 h	CDC Day 30	FiO_2_ 80%: 32/297 (10.8%) FiO_2_ 30%: 32/296 (10.8%)	*NA*	*NA*	*NA*	2–5 ml/kg/h

*O*_*2*_ oxygen, *SSI* surgical site infection, *n* number, *Temp*. temperature (i.e. protocol for maintenance of intraoperative normothermie), *N*_*2*_ nitrogen, *N*_*2*_*O* nitrous oxide, *AA* ambient air, *CDC* Centers for Disease Control and Prevention, *ASEPSIS* Additional treatment, Serous discharge, Erythema, Purulent discharge, Separation of deep tissues, Isolation of bacteria, and prolonged Stay in hospital > 14 days, *RCT* randomized controlled trial.

**Table 2:** Summary of the main characteristics and results of the studies including patients under regional anesthesia.

Study	Country	Design, n	Type of surgery	O_2_ duration in PACU	SSI definition, follow up	SSI, n (%)	Second gas	Antibiotic prophylaxis	Temp	Fluids
Gardella et al.^43^	USA	RCT Monocenter n = 143	Caesarean section under regional anesthesia	2 h	Endometritis or wound infection requiring ATB, Day 14	Mask 15 L/min: FiO_2_ 80%: 17/69 (25%) FiO_2_ 30%: 10/74 (14%) *p* = 0.13	Air	Yes (at cord clamp)	*NP*	*NP*
Scifres et al.^44^	USA	RCT Monocenter n = 585	Caesarean section under regional anesthesia	2 h	Endometritis or wound infection, Day 30	10 L/min (FiO_2_ 80%): 35/288 (12.2%) 2 L/min (FiO_2_ 30%): 26/297 (8.8%) *p* = 0.18	Air	Yes	*NP*	*NP*
Admadé et al.^45^	Panama	RCT Monocenter n = 343	Caesarean section under regional anesthesia	2 h	Wound infection clinical signs, Day 30	FiO_2_ 80%: 9/164 (5.5%) AA: 13/179 (7.3%) *p* = 0.33	Air	Yes	*NP*	*NP*
Duggal et al.^46^	USA	RCT Monocenter n = 831	Caesarean section under regional anesthesia	1 h	CDC (SSI) + Endometritis, Day 45	10 L/min (FiO_2_ 80%): 34/416 (8.2%) 10 L/min (FiO_2_ 30%): 34/415 (8.2%) *p* = 0.89	Air	Yes	*NP*	*NP*
Williams et al.^47^	USA	RCT Monocenter n = 160	Caesarean section under regional anesthesia	2 h	CDC (SSI) + endometritis, Day 42	FiO_2_ 80%: 12/83 (14.5%) FiO_2_ 30%: 10/77 (13.0%) *p* = 0.79	Air	Yes (at cord clamp)	*NP*	*NP*
Fariba et al.^48^	Iran	RCT Monocenter n = 122	Caesarean section under regional anesthesia	6 h	ASEPSIS, Day 14	FiO_2_ 80%: 0/61 FiO_2_ 30%: 1/61 *p* > 0.05	Air	Yes	*NP*	8 ml/kg

*O*_*2*_ oxygen, *SSI* surgical site infection, *n* number, *Temp.* temperature (i.e. protocol for maintenance of intraoperative normothermia), *N*_*2*_ nitrogen, *N*_*2*_*O* nitrous oxide, *AA* ambient air, *CDC* Centers for Disease Control and Prevention, *ASEPSIS* Additional treatment, Serous discharge, Erythema, Purulent discharge, Separation of deep tissues, Isolation of bacteria, and prolonged Stay in hospital > 14 days, *RCT* randomized controlled trial.

In addition, the randomized study by Anthony et al.^18^ was excluded as they assessed a bundle of five measures including 80% FiO_2_ during the surgery and the first 2 postoperative hours as compared with a standard of care using 30% FiO_2_. Indeed, the specific role of high or low FiO_2_ could not be individualized from other measures such as perioperative warming to maintain normothermia or reduction of intravenous fluids during the surgery in this study^18^. Eventually, 30 randomized studies were included in this meta-analysis for a total of 18,055 patients, among which 24 compared high vs. low intraoperative FiO_2_ during general anesthesia (n = 15,871 patients)^19–42^ and 6 during regional anesthesia (n = 2184 patients)^43–48^. High FiO_2_ was 80% in all studies except in Park’s study (FiO_2_ 60%)^37^; and low FiO_2_ was 30% in all studies except in Lin’s study (FiO_2_ 40%)^38^, Pryor’s and Park’s studies (FiO_2_ 35%)^20,37^, Mayank’s study (FiO_2_ 33%)^33^, and Admadé’s study (room air)^45^.

Concerning surgeries performed under general anesthesia, studies mainly included patients undergoing abdominal surgery (exclusively for 18 and mixed with other surgeries for 3 out of the 24 studies) (Table 1). Concerning surgeries performed under loco-regional anesthesia, the 6 studies included caesarean section patients treated with epidural anesthesia (Table 2). SSI was the main judgment criterion in 22 studies^19–22,24–26,28–30,32–35,39,41,43–48^, and a secondary endpoint in the 8 remaining studies^23,27,31,36–38,40,42^.

SSI were diagnosed using the CDC definition^21,24,26–29,31–33,35,36,39,41,42,46,47^, ASEPSIS definition^25,30,34,48^, or other trial-specific definitions^19,20,22,23,37,38,40,43–45^, in respectively 16, 4 and 10 out of the 30 included studies. Confounding factors influencing the incidence of SSI were variously considered. Antibiotic prophylaxis was protocolized in all studies but sometimes incompletely followed. Maintenance of perioperative normothermia was protocolized in only 13/30 studies^19,21–25,27,31–34,36,38^. Amount of perioperative fluid administered and fluid management strategy was protocolized in 9/30 studies^19,21,22,24,29,33,34,40,42^.

### Meta-analysis and sub-group analyses depending on anesthetic modalities

**Figure 2:**
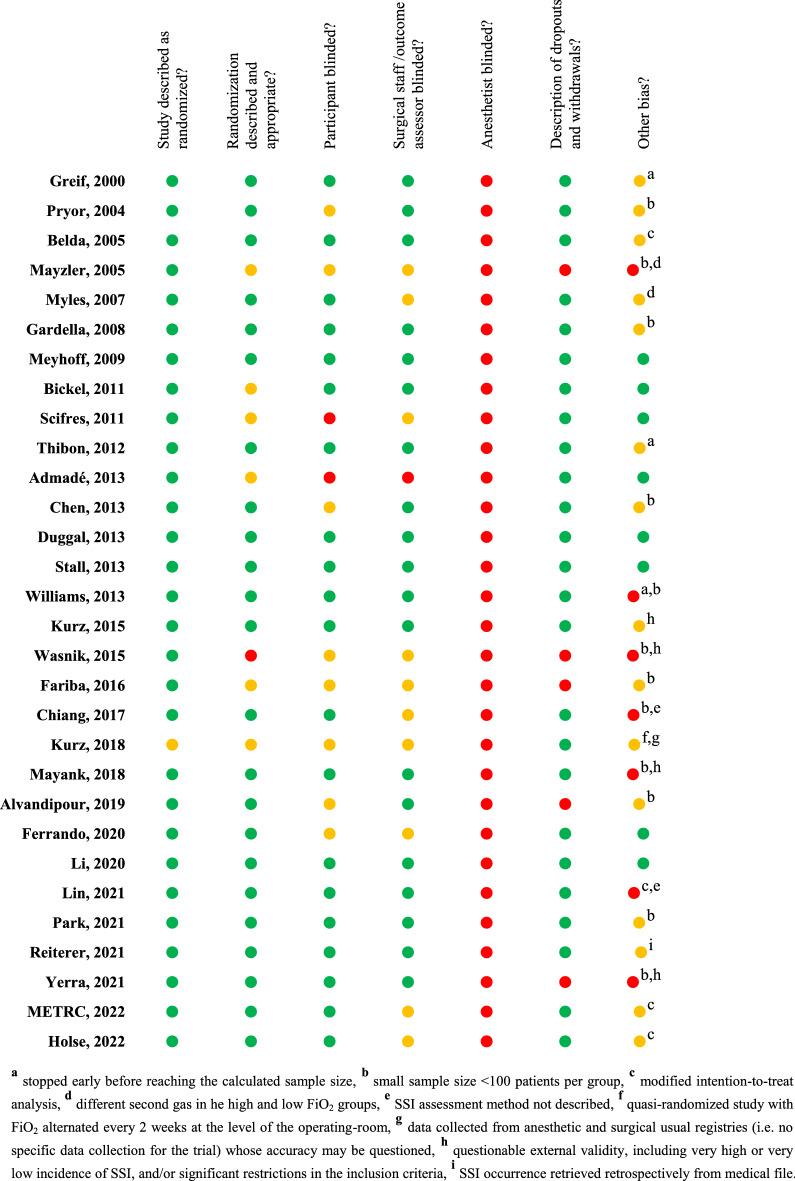
Risk of bias summary for the 30 studies included in the meta-analysis. Green circles represent low risk of bias, yellow circles represent unclear of moderate risk of bias, and red circles represent high risk of bias.

The Oxford quality-scoring system of the 30 studies included in the meta-analysis is summarized in Fig. 2.

**Figure 3:**
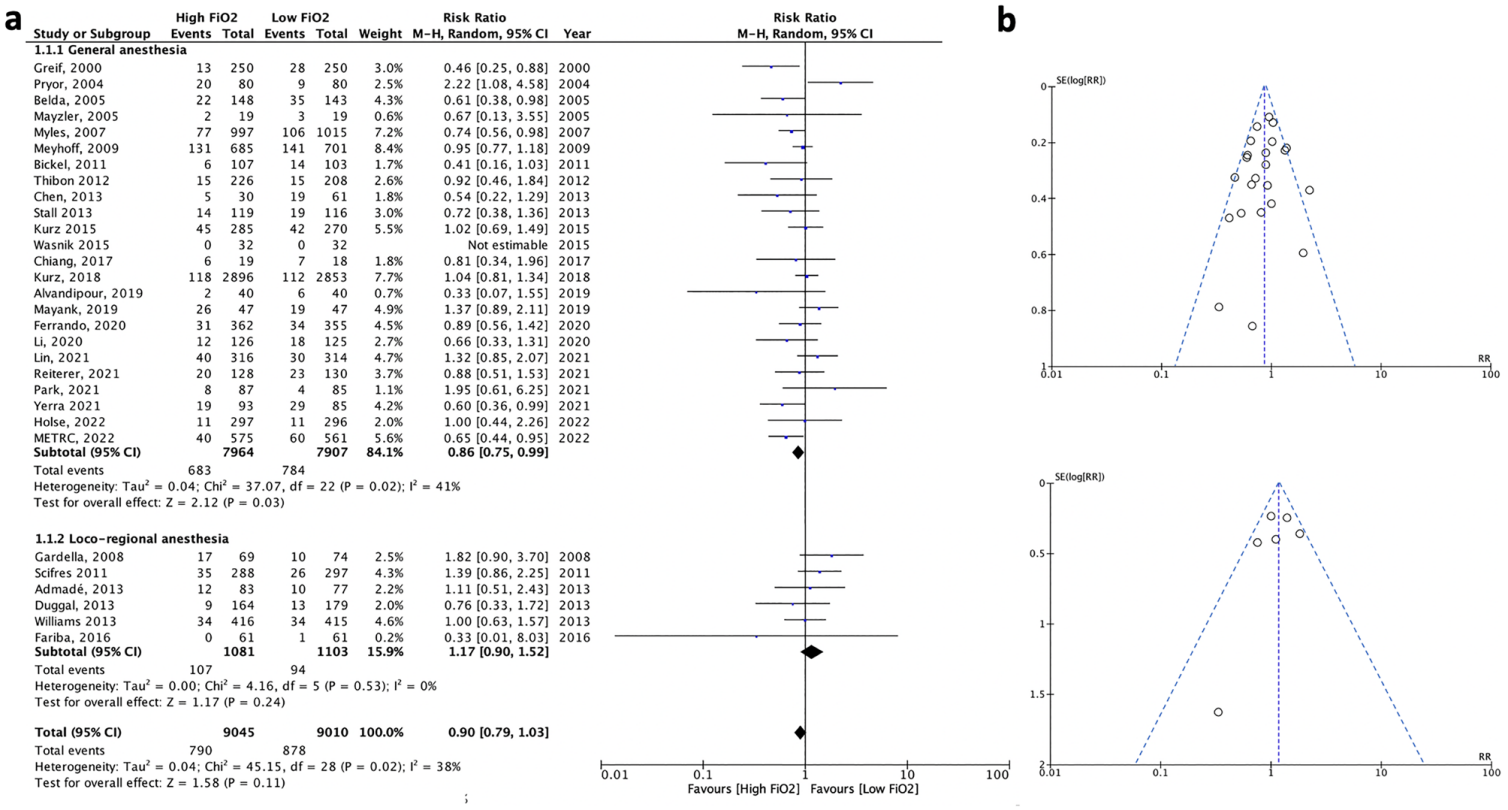
Forest plot analysis of high *vs.* low intraoperative FiO_2_ on the incidence of SSI (**a**), within the subgroups of patients operated under general anesthesia and loco-regional anesthesia; and corresponding funnel plots (**b**). The X-axis of forest plots represents relative risk, and each row on the Y-axis represents an individual study. The blue squares and horizontal lines represent point estimates and corresponding 95% confidence intervals of the individual studies. The black diamonds represent the overall analysis.

Meta-analysis of the 30 studies showed no significant benefit of high FiO_2_ on the prevention of SSI (RR0.90, 95%CI 0.79 to 1.03) (Fig. 3a). There was evidence of heterogeneity (τ^2^ = 0.04, χ^2^ test for heterogeneity *p* = 0.02, I^2^ = 38%). Visual inspection of the funnel-plot showed no clear evidence of publication bias, as confirmed by Egger’s test (Z = − 0.774, *p* = 0.44) and the rank correlation test (Kendall’s τ = − 0.103, *p* = 0.44) (Fig. 3b).

Considering sub-group analyses depending on anesthetic modalities, a moderate benefit was found in patients operated under general anesthesia (RR 0.86, 95%CI 0.75–0.99) (Fig. 3a). There was evidence of heterogeneity (τ^2^ = 0.04, χ^2^ test for heterogeneity *p* = 0.02, I^2^ = 41%). Visual inspection of the funnel-plot showed no clear evidence of publication bias, as confirmed by Egger’s test (Z = − 0.822, *p* = 0.41) and the rank correlation test (Kendall’s τ = − 0.109, *p* = 0.48) (Fig. 3b). According to the GRADE methodology, the overall quality of evidence for prevention of surgical site infection was assessed as low due to biases in individual trials and inconsistency between studies (I^2^ = 41%).

Meta-analysis of the 6 studies that included patients operated on under regional anesthesia showed no significant benefit of high FiO_2_ on the prevention of SSI (RR 1.17, 95%CI 0.90–1.52—Fig. 3a), with good between-study homogeneity (τ^2^ = 0.00, χ^2^ test for heterogeneity *p* = 0.53, I^2^ = 0%). Visual inspection of the funnel-plot showed no clear evidence of publication bias, as confirmed by Egger’s test (Z = − 0.561, *p* = 0.58) and the rank correlation test (Kendall’s τ = − 0.067, *p* = 1.00) (Fig. 3b). According to GRADE methodology, the overall certainty for prevention of surgical site infection was assessed as moderate, taking into account the absence of inconsistency (I^2^ = 0%) but biases in individual studies and the imprecision of the 95%CI around the estimate.

### Sensitivity analyses

#### Second gases

**Figure 4:**
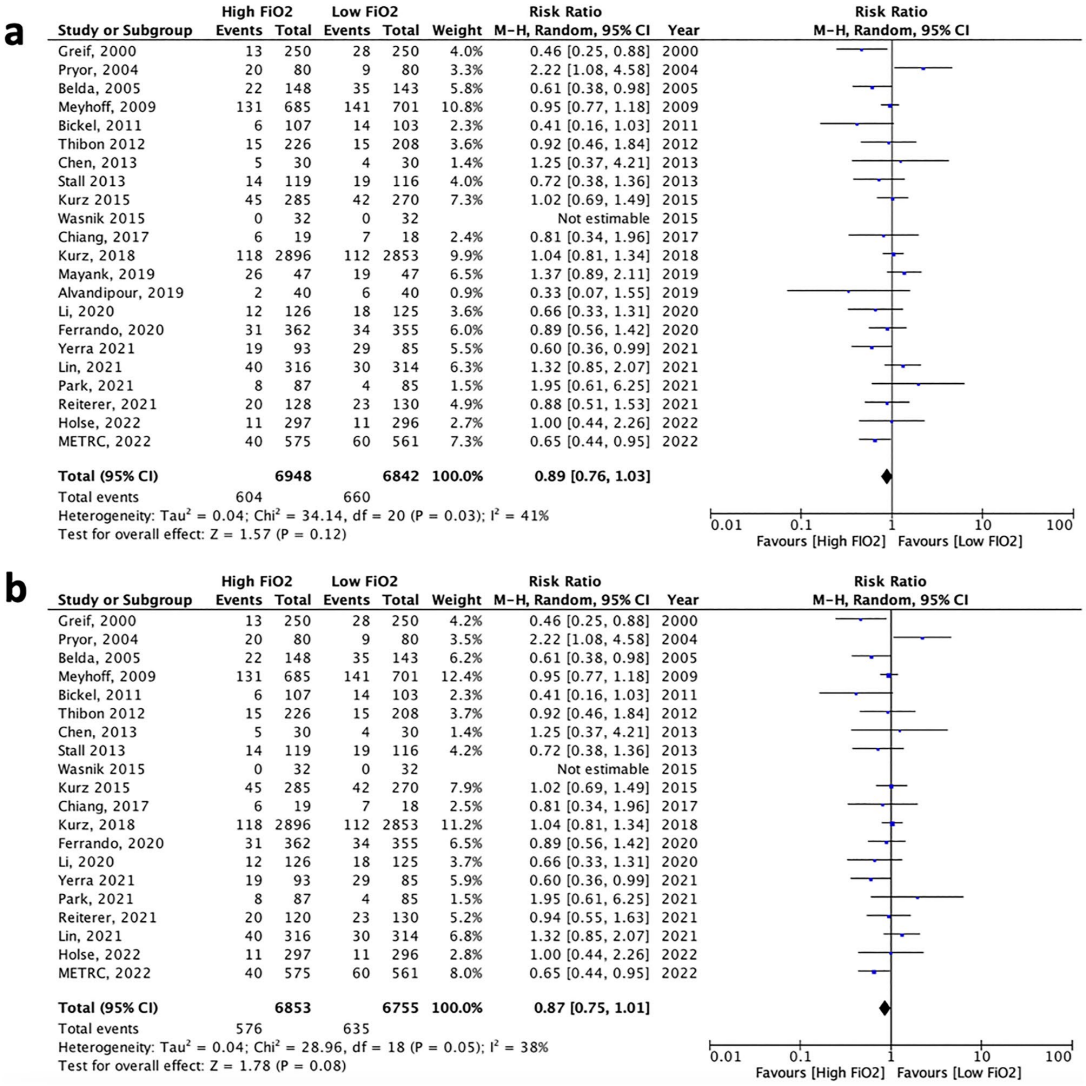
Sensitivity analysis of high versus low intraoperative FiO_2_ on the incidence of SSI in patients operated under general anesthesia restricted to the 22 studies that used the same second gas in both the high and low FiO_2_ group (**a**); and to the 20 studies that did not use nitrous oxide as second gas, neither in the high nor low FiO_2_ groups (**b**).

Considering that it has been suggested that nitrous oxide could impair human immune functions, sensitivity analyses were conducted: (1) on the 22 studies that used the same second gas in both the high and low FiO_2_ groups, i.e. excluding the 2 studies that compared “low FiO_2_ + nitrous oxide” to “high FiO_2_ + nitrogen”^22,23^, and the “low FiO_2_ + nitrous oxide” group of Chen’s study^27^“; showing no significant effect of high intraoperative FiO_2_ (RR 0.89, 95%CI 0.76–1.03—Fig. 4a); and (2) on the 20 studies that did not use nitrous oxide as second gas, neither in the high nor low FiO_2_ groups; showing no significant effect of high intraoperative FiO_2_ (RR 0.87, 95%CI 0.75–1.01—Fig. 4b).

#### Types of surgery

**Figure 5:**
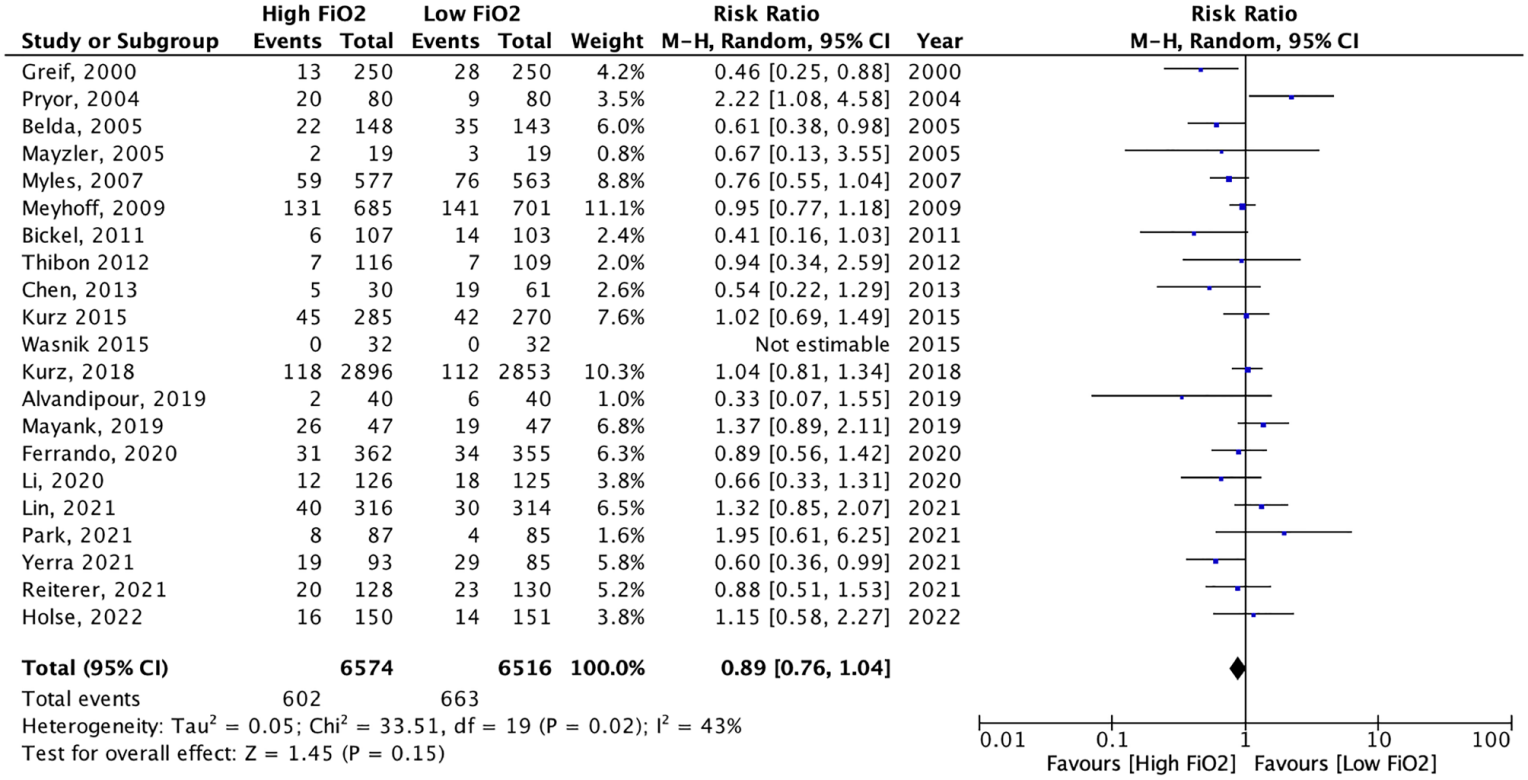
Sensitivity analysis of high *vs.* low intraoperative FiO_2_ on the incidence of SSI in patients operated from abdominal surgery, included in the 18 studies having exclusively included patients operated from abdominal surgeries and in the 3 studies having included mixed surgeries.

Considering that the type of surgery is an important factor associated with the occurrence of SSI, a sensitivity analysis was conducted on the 18 studies having exclusively included patients operated from abdominal surgeries^19–22,24,25,27,29,30,32–40^ and on the subgroup of patients from the 3 studies having included mixed surgeries who were operated from abdominal surgeries, after having obtained additional data regarding these subgroups from the authors^23,26,42^. No significant benefit of high FiO_2_ on the prevention of SSI in abdominal surgery was found (RR0.89, 95%CI 0.76–1.04) (Fig. 5).

#### Types of SSI

**Figure 6:**
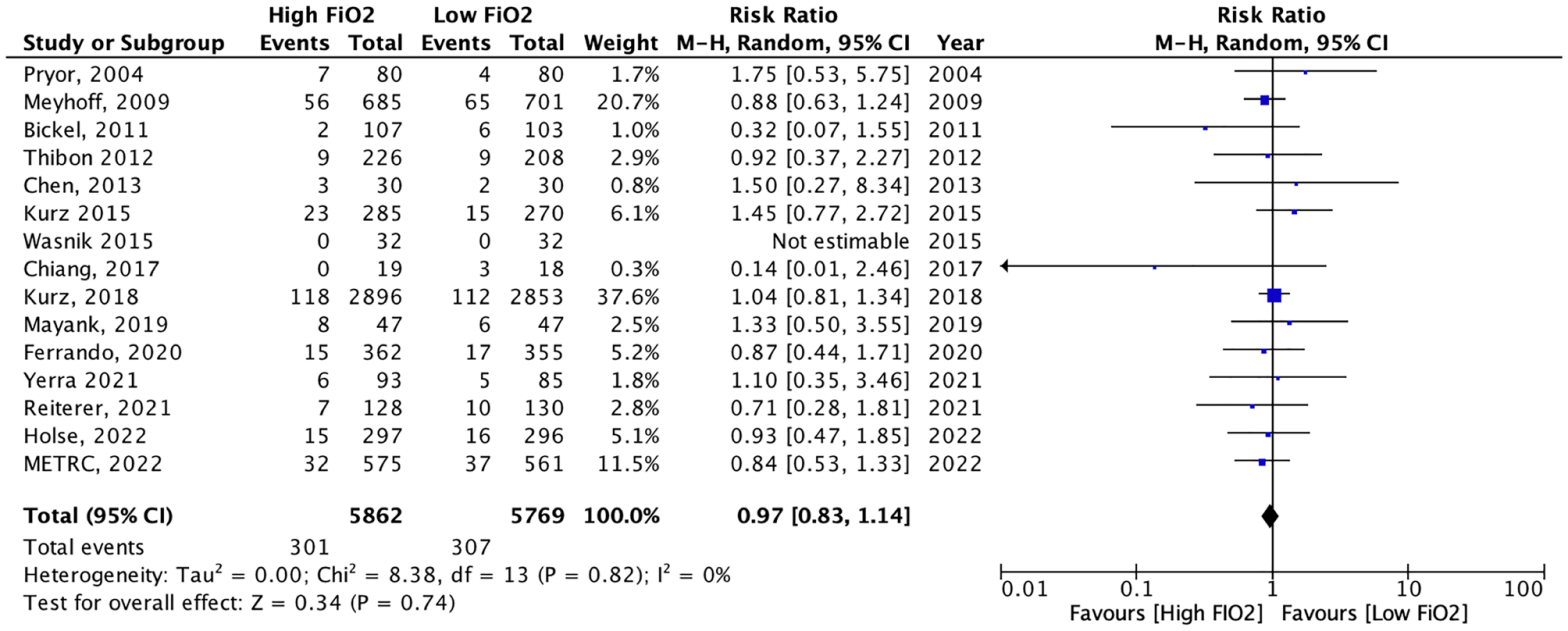
Sensitivity analysis of high versus low intraoperative FiO_2_ on the incidence of deep SSI in patients included in the 15 studies for which data regarding superficial and deep SSI were available or retrieved from the authors.

Considering that superficial SSI, sometimes only treated by local measures of the wound, may be not associated to the same morbidity and mortality than deep SSI, a sensitivity analysis was conducted on the 15 studies for which data on superficial and deep SSI were available or retrieved from the authors. No significant benefit of high FiO_2_ on the prevention of deep SSI was found (RR0.97, 95%CI 0.83–1.14) (Fig. 6).

## Discussion

This updated meta-analysis performed on 30 RCT published between 2000 and July 2022 showed no significant benefit of a high FiO_2_ on the prevention of SSI when considering all types of surgery and anesthetic modalities. This result is even more robust in patients having caesarean section under epidural or spinal anesthesia, leading to recommend that routine administration of supplemental oxygen to these patients should be avoided, considering the absence of any impact on neither maternal nor fetal prognosis^49^. Focusing on patients operated under general anesthesia, a moderate reduction of the incidence of SSI in the high intraoperative FiO_2_ group was observed (RR 0.86, 95%CI 0.75–0.99), however somewhat smaller than that reported by de Jonge et al*.* on studies from 1990 to 2018 (RR 0.80 [0.64–0.99])^8^. The inclusion of the large ENIGMA study in the analysis, whose sample represented more than one third of the total population included in de Jonge’s meta-analysis, may be a source of discussion. Indeed, ENIGMA study, like Mayzler’s study^22^, used nitrous oxide as second gas in the low FiO_2_ group and nitrogen in the high FiO_2_ group, while it has been suggested that nitrous oxide could impair human immune functions and favor SSI^50–52^. However, current evidence does not support any relevant clinical effect of nitrous oxide on SSI occurrence^53^. Consequently, despite an experimental plan not designed originally to assess the role of high vs. low FiO_2_ but rather benefits and harms of nitrous oxide, we chose, as previously done by de Jonge et al. to include Myles’ and Mayzler’s studies in the meta-analysis. One of the advantages of our meta-analysis other the latter is the inclusion of ten new randomized studies published since then^33–42^, representing 4019 additional patients. In addition, we believe de Jonge et al*.* missed the studies by Chiang et al*.* (for patients under general anesthesia)^31^ and Admadé et al*.* (for patients under general anesthesia)^45^, yet published within their inclusion period in 2017 and 2013 respectively. Finally, we believe that Kurz’s controlled cluster trial should have been included as this design comprised protections against biases very close to studies randomized at patient’s level, and provided data based on by far the largest sample of patients. To summarize, our analysis was performed on 30 controlled trials totaling 18,055 patients, compared to 17 studies and 7817 patients in de Jonge’s meta-analysis.

For all that, does this result allow us to conclude definitively that a high intraoperative FiO_2_ is useful to prevent SSI in intubated patients? We believe that not at all. First, our meta-analysis, as did the previous ones, presents substantial heterogeneity making difficult to take the results at face value. Indeed, some studies included very small samples of less than 100 patients^22,27,30,31,33,34^ while others included more than 500 patients^23,24,29,32,35,38,41,42,44,46^. The incidence of SSI was also very different among studies, from a few percent in some studies^26,30,32,42^ to 20% or more in others^21,24,31,33,39^. Differences in the incidence of SSI among studies may also be explained by the heterogeneous control of the confounding risk factors of SSI, such as correct administration of antibiotic prophylaxis, perioperative maintenance of normothermia, amount of fluids infused during the perioperative period, etc. In addition, definitions and times of assessment of SSI may have differed between studies, by using CDC or ASEPSIS definitions or other “home-made” diagnostic criteria; or considering only deep of both superficial and deep SSI. Finally, the surgical site (abdominal vs. extra-abdominal), the surgical approach (laparotomy or laparoscopy), and the indication of surgery (acute vs. planned surgery, carcinologic vs. non-carcinologic surgery) were heterogeneous among studies, while these parameters are known to impact SSI incidence.

Second, beyond the heterogeneity itself, the level of evidence of our meta-analysis, like others on the field, is only low to moderate depending on the considered sub-groups. For patients under general anesthesia, the numerous biases of individual studies (Fig. 1) and the imprecision of the 95%CI around the estimate despite more than 20 studies included in the meta-analysis, led to downgrade the level of evidence. As an example, adding only 5 SSI in the “high FiO_2_ group”, out of a total of more than 680 SSI, makes the confidence interval crossing the identity line, leading to conclude to the absence of significant effect. This highlights the fragility of the conclusions that could be drawn from this unrestricted analysis.

In this context, sensitivity analyses restricted to more homogenous populations may be more informative. Along these lines, no significant effect of a high intraoperative FiO_2_ was found for patients operated from abdominal surgeries under general anesthesia; no more than for patients anesthetized without nitrous oxide; or even for the prevention of deep SSI rather than all types of SSI. We believe that this absence of protective effect in these subgroups of interest strongly reduces the potential interest of systematic high intraoperative FiO_2_. This becomes even more relevant when considering the results of another recent meta-analysis focusing on the effect of high vs. low intraoperative FiO_2_ on respiratory outcomes, which did not report any beneficial effect on clinical outcomes such as hospital length-of-stay or mortality, and on the contrary a higher incidence of postoperative radiographic atelectasis associated with reduced postoperative PaO_2_ values^54^.

Finally, our results and others also demonstrate that meta-analyses are not magic tools able to overcome limits or biases presented by individual studies. In this context, considering the results of well-designed, multicenter, adequately powered randomized controlled trials, using the latest ventilatory and SSI prevention standards should be a more relevant approach than swearing by meta-analyses. In that case, the most recent randomized studies reported no difference on the incidence of SSI with high or low FiO_2_ in patients undergoing general anesthesia. The PROXI study, the largest multicenter randomized controlled trial specifically designed to assess the role of high vs. low intraoperative FiO_2_ on SSI^39^, did not report any reduction of the incidence of SSI with the administration of 80% FiO_2_ during colorectal surgery. Similarly, the recent multicenter randomized iPROVE-O_2_ trial that included 740 patients undergoing major abdominal surgery, ventilated intraoperatively with an evidence-based protective strategy, reported a similar SSI rate between the 30% and 80% FiO_2_ groups^35^. Indeed, 80% FiO_2_ did not reduce postoperative SSI (8.9% vs 9.4%, RR 0.94 95%CI (0.59–1.50)—*p* = 0.90), as none of the secondary outcomes including hospital length-of-stay or short-term mortality. We believe that these results from individual high-quality RCT add further weight and reinforce the conclusion that there is no compelling evidence that high FiO_2_ can improve postoperative patient’s outcome on its own when good SSI prevention practices are properly applied. Consequently, we believe that abrogation of the WHO recommendation on the systematic use of high intraoperative FiO_2_ must be seriously discussed.

## Methods

This meta-analysis followed the Preferred Reporting Items for Systematic Reviews and Meta-Analyses (PRISMA) guidelines. The PRISMA checklist is available as a Supplementary file.

### Registration

A standard protocol was developed and registered prior to literature search on the PROSPERO database on June 1st 2021 (registration number CRD42021258279). The protocol is available as a Supplementary file.

### Search strategy

A search was conducted for studies published between January 1st, 1999 and July, 1st 2022 in MEDLINE (PubMed), CENTRAL (Cochrane), EMBASE (Elsevier) and ClinicalTrials.gov databases. The last search was conducted on July, 5th 2022. The research question was formulated according to the PICO format: in adult patients undergoing general or loco-regional anesthesia (P), does a systematic high intra-operative FiO_2_ (> 50%) (I) lead to reduced incidence of surgical site infection (O) compared to low FiO_2_ (≤ 50%) (C)? Then, the following search equation was designed: ((“Perioperative" [All Fields] OR "intraoperative" [All Fields]) AND ("FiO_2_" [All Fields] OR "inspired oxygen fraction" [All Fields] OR "oxygen concentration" [All Fields]) OR ("anaesthesia" [All Fields] OR "anesthesia" [MeSH Terms] OR "anesthesia" [All Fields] OR "anaesthesias" [All Fields] OR "anesthesias" [All Fields] OR "general anesthesia" [All Fields] OR "general anaesthesia" [All Fields])) AND ("surgical wound infection" [MeSH Terms] OR ("surgical" [All Fields] AND "wound" [All Fields] AND "infection" [All Fields]) OR "surgical wound infection" [All Fields] OR ("surgical" [All Fields] AND "site" [All Fields] AND "infection" [All Fields]) OR "surgical site infection" [All Fields]) OR ("outcomes" [All Fields] OR "adverse effects" [All Fields] OR "adverse events" [All Fields] OR "death" [All Fields]) AND ("pulmonary complications" [All Fields] OR "atelectasis" [All Fields]). Only randomized studies (including quasi-randomized studies and cluster-randomized studies in which the intervention was not randomized at the patient level but by day, week or specific operating theatre) were included in this meta-analysis. In addition, the references of the selected articles were also screened to complete the search. Finally, the PubMed “similar article” and “citing article” functions were used to expand the search.

### Study selection

Two authors (Y.E. and M.G.) independently screened the titles and abstracts retrieved from the systematic search for potential eligibility. In case of discrepancy, the eligibility was discussed with a third author (C.F). To be considered for analysis, publications had to be written in English or in French. When the title and abstract indicated potential eligibility, the full-text article was analysed. The PRISMA flow diagram of study selection is presented in Fig. 1.

### Data extraction and analysis

For each study, a first reviewer extracted the following data: first author, year of publication, study location, type of study, population studied, type of surgery, primary and secondary outcomes selected, and main results. Potential confounding factors that may influence the selected outcomes (for example the perioperative use of antibiotics, the composition of the inspired gas mixture, the use of a protocol to avoid perioperative hypothermia, etc.) were reported. A second reviewer checked independently the extracted data. In case of discrepancy, the data were discussed with a third reviewer and a consensus decision was made. Study sample size and the relevance of the research were considered at the level of each study. Then, the methodological quality of studies was rated with the Oxford quality scoring system considering the SSI outcome^19^.

### Meta-analysis

A quantitative review of the extracted data was made for the judgment criterion (i.e. incidence of SSI). This primary outcome was expressed using the pooled relative risk with its 95% confidence interval (RR 95%CI). A DerSimonian and Laird random-effects model of meta-analysis was used to account for potential clinical and statistical heterogeneity. The χ^2^ test for heterogeneity was computed and the amount of heterogeneity was quantified by the I^2^ statistic. The extent of heterogeneity was evaluated using the between-study variance (τ^2^). Sensitivity analyses including only (1) studies using the same second gas in both the high and low FiO_2_ groups, and (2) studies using nitrogen or room air as second gas in both the high and low FiO_2_ groups (i.e. not including studies that used nitrous oxide as second gas) were planned. Then, sensitivity analyses regarding the type of surgery (abdominal vs. non abdominal), and the type of SSI (deep vs. superficial) were also performed. According to the CDC definition, SSI were considered as “deep SSI” is they were classified as “deep” or “organ/space”.

Eventually, publication bias was evaluated by a visual inspection of funnel plots and assessed with Egger’s regression test and the rank correlation test for funnel plot asymmetry. Statistical analyses were performed using Review Manager (RevMan) 5.4.1 (Cochrane Collaboration, The Nordic Cochrane Centre, Copenhagen, Denmark) and Jamovi 2.0 (The Jamovi Project).

### Quality of evidence

The certainty of the overall evidence for the potential association between the use of high vs. low FiO_2_ and SSI incidence was evaluated using the Grading of Recommendations Assessment, Development and Evaluation (GRADE) methodology, and reported as “very low”, “low”, “moderate” or “high” taking into account study limitations, inconsistency of evidence, indirectness of evidence and reporting bias^55^.

## Supplementary Information

Supplementary Information 1.

Supplementary Information 2.

## Data Availability

All data generated or analyzed during this study are included in this published article and its supplementary information files.
