# Supplementary material for: Updated meta-analysis on intraoperative inspired fraction of oxygen and the risk of surgical site infection in adults undergoing general and regional anesthesia
Source: Sci Rep. 2023 Feb 11;13:2465. doi: 10.1038/s41598-023-27588-2 (PMC9922261; doi:10.1038/s41598-023-27588-2)
Supplement: Supplementary file 2 — Supplementary Information 2. [file 41598_2023_27588_MOESM2_ESM.pdf]

National Institute for Health Research

UNIVERSITY of York
Centre for Reviews and Dissemination

Systematic review

1. * Review title.

Give the title of the review in English

Systematic review followed by a meta-analysis on Intraoperative Inspired Fraction of Oxygen and the risk of Surgical Site Infection

2. Original language title.

For reviews in languages other than English, give the title in the original language. This will be displayed with the English language title.

3. * Anticipated or actual start date.

Give the date the systematic review started or is expected to start.

01/06/2021

4. * Anticipated completion date.

Give the date by which the review is expected to be completed.

30/09/2021

5. * Stage of review at time of this submission.

Tick the boxes to show which review tasks have been started and which have been completed. Update this field each time any amendments are made to a published record.

Reviews that have started data extraction (at the time of initial submission) are not eligible for inclusion in PROSPERO. If there is later evidence that incorrect status and/or completion date has been supplied, the published PROSPERO record will be marked as retracted.

This field uses answers to initial screening questions. It cannot be edited until after registration.

The review has not yet started: No

Review stage	Started	Completed
Preliminary searches	Yes	No
Piloting of the study selection process	No	No
Formal screening of search results against eligibility criteria	No	No
Data extraction	No	No
Risk of bias (quality) assessment	No	No
Data analysis	No	No

Provide any other relevant information about the stage of the review here.

6. * Named contact.

The named contact is the guarantor for the accuracy of the information in the register record. This may be any member of the review team.

Yoann ELMALEH

Email salutation (e.g. "Dr Smith" or "Joanne") for correspondence:

Dr ELMALEH

7. * Named contact email.

Give the electronic email address of the named contact.

yoann.elmaleh@aphp.fr

8. Named contact address

Give the full institutional/organisational postal address for the named contact.

Hopital Tenon, APHP, 4 rue de la Chine, 75020 Paris, France

9. Named contact phone number.

Give the telephone number for the named contact, including international dialling code.

+33(0)781330431

10. * Organisational affiliation of the review.

Full title of the organisational affiliations for this review and website address if available. This field may be completed as 'None' if the review is not affiliated to any organisation.

Hopital Tenon

Organisation web address:

11. * Review team members and their organisational affiliations.

Give the personal details and the organisational affiliations of each member of the review team. Affiliation refers to groups or organisations to which review team members belong. **NOTE: email and country now MUST be entered for each person, unless you are amending a published record.**

Dr Yoann ELMALEH. Hopital Tenon
Dr Marc GARNIER. Hopital Tenon, APHP
Professor Christophe QUESNEL. Hopital Tenon
Dr Charlotte Fasquel. CHU Brest

12. * Funding sources/sponsors.

Details of the individuals, organizations, groups, companies or other legal entities who have funded or sponsored the review.

No funding

Grant number(s)

State the funder, grant or award number and the date of award

13. * Conflicts of interest.

List actual or perceived conflicts of interest (financial or academic).

None

14. Collaborators.

Give the name and affiliation of any individuals or organisations who are working on the review but who are not listed as review team members. **NOTE: email and country must be completed for each person, unless you are amending a published record.**

Professor Olivier Huet. CHU Brest

15. * Review question.

State the review question(s) clearly and precisely. It may be appropriate to break very broad questions down into a series of related more specific questions. Questions may be framed or refined using PI(E)COS or similar where relevant.

- Is the perioperative and/or postoperative administration of high inspired oxygen fraction (FiO2) compared with low FiO2, associated with a decreased risk of postoperative surgical site infections in patients undergoing general anaesthesia ?
- Is the perioperative and/or postoperative administration of high inspired oxygen fraction (FiO2) compared with low FiO2, associated with a decreased risk of postoperative surgical site infections in patients undergoing loco-regional anaesthesia ?
- Is the perioperative and/or postoperative administration of high inspired oxygen fraction (FiO2) compared with low FiO2, associated with short term and/or long term complications in patients undergoing general or loco-regional anaesthesia ?

16. * Searches.

State the sources that will be searched (e.g. Medline). Give the search dates, and any restrictions (e.g. language or publication date). Do NOT enter the full search strategy (it may be provided as a link or attachment below.)

MEDLINE (PubMed), CENTRAL (Cochrane) and ClinicalTrials.gov databases

17. URL to search strategy.

Upload a file with your search strategy, or an example of a search strategy for a specific database, (including the keywords) in pdf or word format. In doing so you are consenting to the file being made publicly accessible. Or provide a URL or link to the strategy. Do NOT provide links to your search **results**.

Search equation

(("perioperative"[All Fields] OR "intraoperative" [All Fields]) AND ("FiO2"[All Fields] OR "inspired oxygen fraction" [All Fields] OR "oxygen concentration" [All Fields]) OR ("anaesthesia"[All Fields] OR "anesthesia"[MeSH Terms] OR "anesthesia"[All Fields] OR "anaesthesias"[All Fields] OR "anesthesias"[All Fields] OR "General anesthesia" [All Fields])) AND ("surgical wound infection"[MeSH Terms] OR ("surgical"[All Fields] AND "wound"[All Fields] AND "infection"[All Fields]) OR "surgical wound infection"[All Fields] OR ("surgical"[All Fields] AND "site"[All Fields] AND "infection"[All Fields]) OR "surgical site infection"[All Fields]) OR("outcomes" [All Fields] OR "adverse effects" [All Fields] OR "adverse events" [All Fields] OR "death" [All Fields]) AND ("pulmonary complications" [All Fields] OR "atelectasis" [All Fields]).

Alternatively, upload your search strategy to CRD in pdf format. Please note that by doing so you are consenting to the file being made publicly accessible.

Do not make this file publicly available until the review is complete

18. * Condition or domain being studied.

Give a short description of the disease, condition or healthcare domain being studied in your systematic review.

Surgical site infections (SSI) are the most common healthcare-associated infections. While advances have been made in infection control practices, including improved operating room ventilation, sterilization methods, barriers, surgical technique, antimicrobial prophylaxis, intraoperative control of hyperglycaemia and hypothermia, SSIs remain a substantial cause of morbidity, prolonged hospitalization, and death. Indeed, SSI is associated with a mortality rate of 3%, and 75% of SSI associated deaths are directly attributable to the SSI. The role of intraoperative FiO2, notably during general anaesthesia is still a matter of debate.

Many definitions of SSI are available. However, they mainly include, within 30 days after surgery, the presence of superficial and/or deep local signs of infection on the skin and subcutaneous tissue around the incision (purulent drainage, localized pain or tenderness, localized swelling, erythema or heat, an abscess or other evidence of infection involving the deep incision, etc.) + isolation of organism(s) identified from an aseptically-obtained specimen.

The CDC and ASEPSIS definition of SSI will be used in this review; however other trial-specific definitions will be considered until they have used the criteria listed above

19. * Participants/population.

Specify the participants or populations being studied in the review. The preferred format includes details of both inclusion and exclusion criteria.

Adult patients operated undergoing general or locoregional anaesthesia, treated intraoperatively and/or immediately postoperatively with either systematic high (50%) or low (?50%) FiO2.

20. * Intervention(s), exposure(s).

Give full and clear descriptions or definitions of the interventions or the exposures to be reviewed. The preferred format includes details of both inclusion and exclusion criteria.

The "intervention" will be defined as the use of a systematic "high" FiO2 (50%) intraoperatively and/or during a period following the surgery not exceeding 24h.

21. * Comparator(s)/control.

Where relevant, give details of the alternatives against which the intervention/exposure will be compared (e.g. another intervention or a non-exposed control group). The preferred format includes details of both inclusion and exclusion criteria.

The "control" group (i.e. comparator) will be defined as the use of a systematic "low" FiO2 (?50%) intraoperatively and/or during a period following the surgery not exceeding 24h. Patients who required a transient increase of their FiO2 due to intra or postoperative desaturation will not be excluded from the analysis.

22. * Types of study to be included.

Give details of the study designs (e.g. RCT) that are eligible for inclusion in the review. The preferred format includes both inclusion and exclusion criteria. If there are no restrictions on the types of study, this should be stated.

We will first consider all studies in English and French languages published from January 1rst, 1999 to June, 1rst 2021, assessing the impact on SSI of high vs. low perioperative FiO2 in adult patients undergoing general or locoregional anesthesia.Then editorials, letters to the editor, abstracts, case reports, animal studies and paediatrics studies will be excluded from the analysis. Finally, randomized controlled trials, quasi-experimental studies, prospective cohort studies, and retrospective comparative studies will be reviewed for inclusion in the systematic review and meta-analysis.

23. Context.

Give summary details of the setting or other relevant characteristics, which help define the inclusion or exclusion criteria.

24. * Main outcome(s).

Give the pre-specified main (most important) outcomes of the review, including details of how the outcome is defined and measured and when these measurement are made, if these are part of the review inclusion criteria.

According to the Grade of Recommendation, Assessment, Development and Evaluation (GRADE) methodology, a preliminary classification of outcomes has been made before starting the review of evidence using a 1–9 numerical scale, in which outcomes rated from 1 to 3 were considered as “low importance
outcomes,” from 4 to 6 as “important but not critical outcomes,” and from 7 to 9 as “critical outcomes”.
Consequently, the main judgement criterion used in our literature review will be rated as follows: surgical site infection incidence (importance 8).

Measures of effect

Please specify the effect measure(s) for you main outcome(s) e.g. relative risks, odds ratios, risk difference,

and/or 'number needed to treat.

incidence (in percentage) in both the intervention and control groups; relative risk of SSI occurrence

25. * Additional outcome(s).

List the pre-specified additional outcomes of the review, with a similar level of detail to that required for main outcomes. Where there are no additional outcomes please state ‘None’ or ‘Not applicable’ as appropriate to the review

Secondary criteria = mortality at 30 days (importance 9), adverse respiratory events (importance 7), adverse cardiovascular events (importance 7), length of postoperative stay (importance 6) and incidence of postoperative nausea and vomiting (importance 4).

Measures of effect

Please specify the effect measure(s) for you additional outcome(s) e.g. relative risks, odds ratios, risk difference, and/or 'number needed to treat.

incidence (in percentage) in both the intervention and control groups; relative risk of secondary outcomes occurrence

26. * Data extraction (selection and coding).

Describe how studies will be selected for inclusion. State what data will be extracted or obtained. State how this will be done and recorded.

Data will be extracted from published reports, protocols, and registers by two independent reviewers using a standardized data extraction form. The data extraction sheet will be tested on 20 articles. Two authors will examine each title and abstract identified to exclude irrelevant reports. Two authors will examine full text of eligible articles independently. A comparison across studies, checking author names, treatment comparisons, sample sizes, and outcomes, will be performed to avoid duplicates and compilations of data from several reports of the same study. In case of a discrepancy, a consensus decision was made between the two reviewers.

For each study, a first reviewer will extract the following data: first author, year of publication, study location, type of study, population studied, type of surgery, primary and secondary outcomes selected, and main results. Potential confounding factors that may influence the selected outcomes (for example the perioperative use of antibiotics, the composition of the inspired gas mixture, etc.) will be reported. A second reviewer will check independently the extracted data.

Study sample size and the relevance of the research will be considered at the level of each study. Then, the methodological quality of RCTs will be rated with the Oxford quality scoring system. The analysis will be performed according to decreasing hierarchical prioritization of data from meta-analyses of randomised controlled trials (RCTs) or individual RCTs to observational studies.

27. * Risk of bias (quality) assessment.

State which characteristics of the studies will be assessed and/or any formal risk of bias/quality assessment tools that will be used.

Based on the results of the systematic review, a meta-analysis of RCTs and/or quasi-randomized studies

evaluating the benefit-risk ratio of a high perioperative FiO2 ratio in patients under general or locoregional anaesthesia will be performed. Each study will be assessed for methodological quality prior to inclusion in the meta-analysis using the Cochrane Collaboration tool (Rob tool 2).

28. * Strategy for data synthesis.

Describe the methods you plan to use to synthesise data. This **must not be generic text** but should be **specific to your review** and describe how the proposed approach will be applied to your data. If meta-analysis is planned, describe the models to be used, methods to explore statistical heterogeneity, and software package to be used.

A descriptive analysis of the studies included in the review will be carried out. Quantitative variables will be described as means and standard deviations. Number and percentages will be used to describe categorical variables.

A meta-analysis of RCTs and/or quasi-randomized studies evaluating the association of SSI with perioperative FiO2 in patients undergoing general or locoregional anaesthesia will be performed. This primary outcome will be expressed using the pooled relative risk with its 95% confidence interval (RR 95%CI). The efficiency index used for our quantitative judgement criteria will be the difference in means or standardized difference in means. The overall effect (summary measure) will be estimated using a random effect model (DerSimonian and Laird). Heterogeneity will be assessed by 1/ a visual inspection of forest plots, 2/ the Cochran Q test, and 3/ the I^2 index. The extent of heterogeneity will be evaluated using the between-study variance (tau^2). Statistical analyses will be performed using Review Manager (RevMan) 5.3 (The Nordic Cochrane Centre, The Cochrane Collaboration, 2014).

29. * Analysis of subgroups or subsets.

State any planned investigation of ‘subgroups’. Be clear and specific about which type of study or participant will be included in each group or covariate investigated. State the planned analytic approach.

NA

30. * Type and method of review.

Select the type of review, review method and health area from the lists below.

Type of review

Cost effectiveness
No

Diagnostic
No

Epidemiologic
No

Individual patient data (IPD) meta-analysis
No

Intervention
No

Living systematic review
No

Meta-analysis
Yes

Methodology
No

Narrative synthesis
No

Network meta-analysis
No

Pre-clinical
No

Prevention
No

Prognostic
No

Prospective meta-analysis (PMA)
No

Review of reviews
No

Service delivery
No

Synthesis of qualitative studies
No

Systematic review
Yes

Other
No

Health area of the review

Alcohol/substance misuse/abuse
No

Blood and immune system
No

Cancer
No

Cardiovascular
Yes

Care of the elderly
No

Child health
No

Complementary therapies

No

COVID-19
No

Crime and justice
No

Dental
No

Digestive system
No

Ear, nose and throat
No

Education
No

Endocrine and metabolic disorders
No

Eye disorders
No

General interest
No

Genetics
No

Health inequalities/health equity
No

Infections and infestations
Yes

International development
No

Mental health and behavioural conditions
No

Musculoskeletal
No

Neurological
No

Nursing
No

Obstetrics and gynaecology
No

Oral health
No

Palliative care
No

Perioperative care
Yes

Physiotherapy
No

Pregnancy and childbirth
No

Public health (including social determinants of health)
No

Rehabilitation
No

Respiratory disorders
Yes

Service delivery
No

Skin disorders
No

Social care
No

Surgery
No

Tropical Medicine
No

Urological
No

Wounds, injuries and accidents
No

Violence and abuse
No

31. Language.

Select each language individually to add it to the list below, use the bin icon to remove any added in error.
English
French

There is not an English language summary

32. * Country.

Select the country in which the review is being carried out. For multi-national collaborations select all the countries involved.

France

33. Other registration details.

Name any other organisation where the systematic review title or protocol is registered (e.g. Campbell, or The Joanna Briggs Institute) together with any unique identification number assigned by them. If extracted data will be stored and made available through a repository such as the Systematic Review Data Repository (SRDR), details and a link should be included here. If none, leave blank.

34. Reference and/or URL for published protocol.

If the protocol for this review is published provide details (authors, title and journal details, preferably in Vancouver format)

Add web link to the published protocol.

Or, upload your published protocol here in pdf format. Note that the upload will be publicly accessible.

No I do not make this file publicly available until the review is complete

Please note that the information required in the PROSPERO registration form must be completed in full even if access to a protocol is given.

35. Dissemination plans.

Do you intend to publish the review on completion?

Yes

Give brief details of plans for communicating review findings.?

36. Keywords.

Give words or phrases that best describe the review. Separate keywords with a semicolon or new line. Keywords help PROSPERO users find your review (keywords do not appear in the public record but are included in searches). Be as specific and precise as possible. Avoid acronyms and abbreviations unless these are in wide use.

inspired oxygen fraction, FiO2, surgical site infection, perioperative oxygen, respiratory complications,

mortality.

37. Details of any existing review of the same topic by the same authors.

If you are registering an update of an existing review give details of the earlier versions and include a full bibliographic reference, if available.

Fasquel C, Huet O, Ozier Y, Quesnel C, Garnier M. Effects of intraoperative high versus low inspiratory

oxygen fraction (FiO2) on patient's outcome: A systematic review of evidence from the last 20 years.

Anaesth Crit Care Pain Med. 2020 Dec;39(6):847-858. doi: 10.1016/j.accpm.2020.07.019. Epub 2020 Oct 7.

PMID: 33038560.

38. * Current review status.

Update review status when the review is completed and when it is published.New registrations must be ongoing so this field is not editable for initial submission.
Please provide anticipated publication date

Review_Ongoing

39. Any additional information.

Provide any other information relevant to the registration of this review.

40. Details of final report/publication(s) or preprints if available.

Leave empty until publication details are available OR you have a link to a preprint (NOTE: this field is not

editable for initial submission). List authors, title and journal details preferably in Vancouver format.

Give the link to the published review or preprint.
